# Validity and reliability of an protocol of the stomatognathic and postural system evaluation for patients with obstructive sleep apnea: a cross-sectional study

**DOI:** 10.3389/fphys.2025.1649593

**Published:** 2025-09-30

**Authors:** Marta Guijarro-Herraiz, Natalia M. Arias Palencia, Maria Figueroa Mayordomo, Rocío Palomo Carrión, Blanca Notario Pacheco

**Affiliations:** 1 Faculty of Nursing, Physiotherapy and Occupational Therapy, University of Castilla-La Mancha, Toledo, Spain; 2 Department of Physiotherapy, Faculty of Health Sciences, Universidad Europea de Valencia, Valencia, Spain; 3 Department of Nursing, Faculty of Physiotherapy and Nursing, Physiotherapy and Occupational Therapy, ImproveLab Research Group, University of Castilla-La Mancha, Toledo, Spain; 4 Department of Nursing, Faculty of Nursing of Cuenca, Physiotherapy and Occupational Therapy, University of Castilla-La Mancha, Cuenca, Spain; 5 Faculty of Education of Cuenca, University of Castilla-La Mancha, Cuenca, Spain

**Keywords:** obstructive sleep apnea, myofunctional therapy, sleep apnea, postural reeducation, stomatognathic system, oropharyngeal exercises

## Abstract

**Introduction:**

Myofunctional therapy has been used for years as an intervention with high effectiveness in cases of Obstructive Sleep Apnea (OSA). Until now, little importance has been given to postural attitude or morphotype), even though these can modify the state in which we find the musculature and fascial system of the entire stomatognathic system. The objective if this cross-sectional study is to analyse the validity and reliability of a stomatognathic and postural systems assessment protocol for patients with OSA.

**Methods:**

This is a cross-sectional study that uses new observational and measurement parameters (the postural attitude or morphotype, the position of the hyoid bone, potential temporomandibular joint disorders and mobility of the spheno-basilar joint), were assessed in 105 adult subjects presenting with symptoms associated with obstructive sleep apnea (OSA). All subjects were evaluated using the same guidelines and standardized tests. Participants ranged in age from 23 to 83 years and volunteered to take part in the study. The assessment followed a specific protocol encompassing two main components (A) postural analysis and (B) specific evaluation of the stomatognathic system. All measurements were conducted by the same examiner (MMGH), thereby minimizing interobserver bias.

**Results:**

Criterion validity was assessed using two Spearman correlation tests. The first examined the correlation between the final protocol score and the apnea-hypopnea index (AHI), yielding a value of 0.082. The second analysis assessed the correlation between the diagnostic results from sections A and B of the protocol, with correlation coefficients of 0.824 and 0.907 respectively. Concurrent validity was evaluated using Spearman correlation between the final protocol score and several variables: apnoea–hypopnoea index, Epworth Sleepiness Scale, physical activity, waist circumference, and neck circumference. All correlations were statistically significant (p < 0.01; p < 0.05). An ANCOVA was conducted to examine mean differences in variables across categories of the final protocol score, controlling for age and sex. Internal consistency of the scale was assessed using Cronbach’s alpha, which yielded a coefficient of 0.926. Reproducibility of the protocol was evaluated using the standardised intraclass correlation coefficient (ICC).

**Conclusion:**

The specific protocol for the evaluation of the stomatognathic and postural system for OSA (SPOSAP) is a valid and reliable instrument for the screening of the pathology. We believe that the validation of this protocol may facilitate the identification of undiagnosed patients presenting with compatible symptomatology, and broaden the range of treatment options currently available.

## Introduction

Obstructive sleep apnea (OSA) is defined as the recurrent occurrence of total or partial obstruction of the upper airway (UA), caused by a range of multifactorial alterations of morpho-physiological, functional, and/or polygenic origin. In 2005, the Spanish Society of Pulmonology and Thoracic Surgery (SEPAR) reached a multidisciplinary consensus to refer to this condition as Sleep Apnea–Hypopnea Syndrome (SAHS), to explicitly include hypopneas given their comparable clinical impact to apneas. However, the 2022 update recommended a return to the simplified term *obstructive sleep apnea (OSA)* (Mediano et al., 2022). OSA leads to functional changes in multiple anatomical regions, with the pharyngeal airway being one of the most affected (Diaféria et al., 2017). These anatomical alterations disrupt the function of pharyngeal musculature and the tongue, causing recurrent breathing pauses. Partial airway obstruction results in hypopneas, whereas complete obstruction leads to apneas (Mediano et al., 2019a). These events are characterized by an imbalance between UA patency and the tone of pharyngeal dilator muscles, resulting in compromised neuromuscular responses and determining OSA severity according to the number of episodes per hour (Oksenberg et al., 2006). Diagnosis is established through polysomnography (PSG), with continuous positive airway pressure (CPAP) being the first-line treatment (Santín et al.). Untreated or non-compliant patients are at increased risk of associated comorbidities, including: (a) cardiovascular disease, (b) difficult-to-control hypertension, (c) coronary artery disease, (d) congestive heart failure, (e) arrhythmias, and (f) stroke. During wakefulness, individuals with OSA exhibit increased genioglossus activity as a compensatory mechanism to prevent airway collapse. However, in most cases, the obstruction occurs at the level of the soft palate. Reduced palatal muscle activity increases UA resistance and contributes to the onset of apneas and hypopneas. This is exacerbated by alveolar hypoventilation, a common feature in OSA (Mediano et al., 2019b). Upper airway patency depends on the coordinated function of the UA dilator muscles and the diaphragm. The inspiratory collapse driven by negative intrathoracic pressure is counteracted by the activity of these dilator muscles, particularly the genioglossus, whose contraction facilitates anterior displacement of the tongue and increases pharyngeal airway diameter. Genioglossus hypotonia, conversely, leads to posterior displacement of the tongue and airway collapse.

Sleep and wakefulness are regulated by both homeostatic and circadian mechanisms. During sleep, respiratory changes include decreased respiratory rate, reduced alveolar ventilation, lower tidal volume, changes in blood gas concentrations, and reduced tone and reflex activity in respiratory and UA muscles. These changes predispose the airway to collapse, leading to partial or complete airflow interruption (Richter et al., 1997). OSA is also associated with metabolic dysregulation, impaired glucose control, and increased risk of diabetes (Murayama et al., 2011). Sleep disturbances affect neuroendocrine function, contributing to elevated leptin levels (associated with increased appetite), heightened pro-inflammatory cytokines (e.g., TNF-α and IL-1β), and reduced insulin sensitivity, resulting in impaired glycemic regulation (Weibal et al., 1995; Bierwolf et al., 1997; Durmer and Dinges, 2005). Furthermore, numerous studies have demonstrated that sleep posture influences the apnea–hypopnea index (AHI), with events occurring more frequently in the supine position compared to lateral positions (Murayama et al., 2011; Srijithesh et al., 2019; De Felicio and Pimenta, 2008). This supports the hypothesis of a relationship between body posture and both the incidence and severity of OSA. In this context, authors such as De Felicio (De Felicio and Pimenta, 2008; Folha et al., 2015) and Gigoski de Miranda (De Miranda et al., 2019) have proposed evaluations targeting patients with symptoms of OSA, focusing on the presence of specific anatomical and functional traits within the stomatognathic and orofacial systems. Their goal is to identify shared parameters that may be associated with OSA pathophysiology.

In line with these findings, several studies have demonstrated a direct relationship between the stomatognathic system and body posture, underscoring its key role in postural control in coordination with the somatosensory system. The stomatognathic system comprises various anatomical structures, including the mandible, maxilla, dental arches, soft tissues (such as salivary glands, nerves, and blood vessels), the temporomandibular joint (TMJ), and the masticatory muscles. These components work in an integrated manner to support essential functions, including those involved in postural regulation (Cuccia and Caradonna, 2009).

The TMJ, in particular, is structurally and functionally connected to the cervical spine through muscular and ligamentous pathways, forming the cranio–cervico–mandibular complex. Dysfunction within this complex has been associated with postural deviations, often related to dental occlusion and involving the trigeminal nerve, which plays an active role in the regulation of body posture (Nakahara et al., 2004). For instance, changes in mandibular position and trigeminal nerve input have been linked to alterations in the plantar pressure center through proprioceptive mechanisms, as well as to variations in sternocleidomastoid muscle activity depending on mandibular alignment.

On the other hand, dynamic postural control is achieved through the coordination of proprioceptive, visual, and auditory inputs, along with muscular activity, joint movement, postural reactions to various stimuli, and reflexes that regulate postural tone. This control involves the coordinated function of the vestibulo-labyrinthine system, which maintains vertical alignment, and the oculomotor system, which ensures horizontal gaze stability. The body’s spatial alignment and response depend on three planes: (1) the bipolar plane (horizontal positioning of the eyes), (2) the acoustic plane (perpendicular orientation of the semicircular canals), and (3) the occlusal plane (pathological forward or backward head positioning), all integrated and regulated by the central nervous system (Rodríguez et al., 2004). Several authors have linked postural alterations with the occlusal system and the temporomandibular joint (TMJ) and have described specific postural dysfunction patterns. Simons and Travell (Simons et al., 2002) referred to this condition as forward head posture, while others, such as Janda (Janda, 1984), described it as the proximal crossed syndrome. Forward head position, orofacial muscular hypotonia, improper positioning of the hyoid bone, and functional and physiological alterations of the tongue are among the key factors analyzed in their studies. Other authors, including Kondo (Kondo, 2004) and Tingey (Tingey et al., 2001), have also published research supporting this association. Kondo concluded that manual therapy and postural reeducation techniques can produce changes in TMJ position and occlusion type, and that intervention in either area affects the other. Tingey investigated variations in interdental contacts, TMJ position, and occlusion under different postural conditions, finding significant differences when evaluating these structures in upright standing posture with and without support. Bollhalder (Bollhalder et al., 2013) also supports these findings in his study. Several studies have addressed the correlation between the stomatognathic system and the musculoskeletal system, highlighting how alterations in the tooth–mandible–tongue complex may influence postural alignment. The findings are promising and suggest that the primary mediator of this association is the trigeminal nerve. Numerous anatomical connections have been described between the trigeminal system and neural structures involved in postural control. The mesencephalic nucleus of the trigeminal nerve, in particular, is a sensory nucleus with unique characteristics (Martines et al., 2015; Patti et al., 2016). Regarding muscular strength and the fascial system, studies such as that by Oh et al. have demonstrated that stomatognathic alignment exercises improve temporomandibular joint (TMJ) function. A stomatognathic alignment exercise program was implemented, incorporating TMJ and cervical mobility exercises along with postural correction. By the end of the study, all parameters related to cervical mobility and mouth opening had improved significantly. The results of this pilot study suggest that mandibular position may influence physical performance, with potential practical applications in sports and exercise sciences (Patti et al., 2016).

This study takes an expanded approach to other evaluations, considering postural alterations as one of the most important parameters to be assessed, within those that can affect the SS, both in terms of the existence of the disease and the multidisciplinary approach to treatment. We have included three new measurement variables in the protocol: morphotyping, hyoid bone position and assessment of spheno-basilar joint mobility. The subject is evaluated as a whole, and different anatomical alterations are related to each other.

The main objective of the study is to assess the validity and reliability of a specific evaluation protocol for the stomatognathic and postural system (SPOSAP) for patients presenting symptoms compatible with OSA and to use this protocol as a screening tool in this type of population. The SPOSAP expands on the assessments of the SS in relation to OSA published so far, offering a more comprehensive perspective on all possible morphophysiological alterations present, giving special relevance to the subject’s posture and its alterations. These alterations could have repercussions both on the identification of risk factors related to the pathology and on a different approach to intervention. It could be proposed to approach the subject in a more comprehensive way, including in the Myofunctional Therapy (MT) treatment the work on the muscle chains, which are crucial regarding static and dynamic posture.

## Methods

### Study design and participants

This cross-sectional study was conducted on a total of 105 adult subjects, both male and female, aged between 23 and 83 years. Multiple recruitment strategies were employed to enrol participants in the study. First, a public announcement was issued to the general population, inviting individuals to participate provided they met the predefined inclusion and exclusion criteria. In this case, a preliminary telephone screening was conducted to exclude individuals who did not meet the eligibility requirements. Those who qualified were then scheduled for an in-person appointment, during which the full evaluation protocol was carried out. Additionally, the research committee of the XXXX Health Area (where the study was conducted) was contacted. The committee used its databases to identify individuals who met the eligibility criteria, who were then contacted. Moreover, infographics were placed in key areas of the XXXX Regional Hospital—the only referral hospital in the province—including the Pneumology, Neurophysiology, and General Medicine departments. These materials aimed to draw the attention of individuals who either identified with symptoms characteristic of the condition or had already been diagnosed. Contact information was included in the infographic to facilitate participation. All included subjects manifested symptoms related to OSA and/or were diagnosed according to the International Classification of Diseases ICD-10 criteria, where apnea is represented under code G47.33. All participants were informed about the study procedure and the use of their data and have signed the corresponding consent form.

Participants were included if they were over 18 years of age and exhibited at least two of the following symptoms for a duration of at least 6 months, regardless of whether they had a confirmed diagnosis of obstructive sleep apnea (OSA): persistent snoring; bruxism or a sensation of grinding or clenching the teeth (during the day, at night, or both); fragmented sleep; and/or daytime fatigue. Exclusion criteria comprised the presence of chronic spinal or cranial injuries, active tumours, or the regular use of medications such as analgesics, anaesthetics, psychiatric drugs, or muscle relaxants, as well as other substances that could interfere with sleep, including anxiolytics, antidepressants, antipsychotics, antiepileptics, or related agents. Participants were also excluded if they were unable to understand or complete the evaluation procedures, or if they had limitations that prevented them from engaging in physical activity or performing activities of daily living.

### Sampel size estimation

The sample size was estimated based on the main objective of the study: to assess the validity of the SPOSAP protocol as a screening tool in a population presenting symptoms compatible with obstructive sleep apnea (OSA). For this purpose, the apnea-hypopnea index (AHI) was used as the reference standard, with a classification of OSA risk (low, medium and high) based on standard clinical cut-off points. A minimum clinically relevant difference of at least three points in the Epworth Sleepiness Scale (ESS) between groups was considered, with an estimated standard deviation of 3.5 points, a confidence level of 95%, and a statistical power of 80%. Under these conditions, the minimum required sample size was estimated to be at least 30 participants per group. Ultimately, the total sample included 105 subjects (n = 26 at low risk, n = 39 at medium risk, and n = 40 at high risk of OSA), which ensured sufficient power to detect between-group differences through an ANCOVA model adjusted for age and sex.

### Measurement

All participants were evaluated by single research (XXX) between March and November 2023. Three additional researchers participated in the collection and organization of the recorded data (XXX). To ensure methodological rigor, all variables were measured using standardized procedures. Sociodemographic (age, gender, occupation), clinical (blood pressure, BMI, waist circumference, neck circumference), and behavioral (physical activity according to WHO criteria and IPAQ (International Physical Activity Questionnaire 2022) data were collected through structured interviews and validated instruments. Potential confounding variables were identified based on prior evidence regarding their association with obstructive sleep apnea (OSA) and its severity. These included age, sex, BMI, blood pressure status, and physical activity level. While participant groups (diagnosed vs. non-diagnosed) showed no statistically significant differences in most of these variables, possible confounding effects were taken into account during analysis. Specifically, stratified comparisons and multivariable statistical techniques were used where appropriate to minimize bias and isolate the association between the condition and relevant clinical indicators (e.g., ESS score, AHI). This approach helped ensure that the observed associations were not unduly influenced by background characteristics. Data collection included the following measurements:

Sociodemographic variables: Age, gender, and occupation.

### Anthropometric variables

#### Weight, height, and body mass index (BMI)

During the interview, weight and height values are taken from the participants. Body weight and height were measured using a calibrated digital scale (OMRON BF511). and stadiometer, respectively, with participants barefoot and wearing light clothing. BMI was calculated as weight (kg) divided by height squared (m^2^).

#### Waist circumference (WC)

To measure this parameter, we used the subject’s iliac crests and the last floating rib on each side as references. A mark was made with a dermal pencil at the midpoint of this distance on both sides, and a flexible measuring tape was passed through these two points. The waist circumference measurement was taken with the subject in a relaxed breathing position.

#### Neck circumference (NC)

To measure this parameter, a flexible measuring tape was used to measure around the neck with the thyroid cartilage as a reference, placing the head in a neutral and relaxed breathing position.

#### Blood pressure

Systolic and diastolic blood pressure were measured using a validated automatic sphygmomanometer (OMRON X3 Comfort) after the participant had rested for at least 5 min in a seated position. Hypertension was classified according to current clinical guidelines. the subject sat with the arm at the same height as the heart and without any clothing that might have compressed the upper limb when measuring. The subject was asked not to move or speak during the time taken to measure blood pressure.

#### Physical activity (PA)

We use the IPAQ questionnaire to determine the level of adherence to physical activity. In this questionnaire, the subject is asked a series of questions about the frequency, duration and intensity of physical activity performed in the last 7 days (moderate to intense exercise), finally classifying them as high, moderate, or low/inactive according to the degree of activity and the World Health Organization (WHO) recommendations (WHO guidelines on physical activity, 2020).

#### Sleep-disordered breathing

The Apnea-Hypopnea Index (AHI) was obtained through overnight polysomnography or validated home sleep apnea testing, depending on availability. OSA was classified as mild (5 ≤ AHI <15), moderate (15 ≤ AHI <30), or severe (AHI ≥30), following established clinical criteria according to the ICD-10 criteria: G47.33.

#### Sleepiness

By the Epworth Sleepiness Scale (ESS) questionnaire. The ESS is one of the most widely used scales to assess the impact that OSA may have, in our study we used the scale validated in Spanish (Chiner et al., 1999; Johns, 1991) It will help the patient and the healthcare professional have an initial approach to the diagnosis of different sleep-related breathing disorders by measuring daytime sleepiness. The patient estimates the likelihood (0-never; 1-slight; 2-moderate; 3-high) of falling asleep in eight different situations. Depending on the total score, which can range from 0 to 24, the degree of sleepiness and the possible diagnosis will be determined: snoring, OSA, narcolepsy, hypersomnia, or insomnia, among others. The ESS is not specific to OSA, so it is important to consider that in the Spanish population, scores above 11 are considered pathological, and other sleep disorders must be ruled out. It is also important to note that the Epworth Scale measures daytime sleepiness and provides an approximation of its possible origin, which can help evaluate the impact of OSA on the patient’s quality of life. However, it is also possible for patients with severe OSA to not experience daytime sleepiness and therefore not present a high value on this test (Johns, 1991).

To control for potential confounding variables—such as age, sex, BMI, blood pressure status, and physical activity level—these factors were assessed *a priori* based on existing literature. Although no significant differences were observed between groups in most of these variables, their potential influence was considered during statistical analysis through stratification and multivariable adjustment, as appropriate. This methodological approach aimed to reduce bias and strengthen the internal validity of the study.

#### Comorbidities

The subject’s most important pathological history was also collected, especially those related to cardiovascular and endocrine pathologies and hypertension. These data were obtained through direct questions in the clinical interview and were confirmed by the medical reports provided by the subjects. The main pathologies recorded are cardiovascular, the most frequent being arterial hypertension, cerebrovascular events, mainly stroke or transient ischemic attack, and metabolic disorders, the most important of which is diabetes.

The SPOSAP is divided into two main areas A) Postural system analysis: 1) evaluation of the morphotype according to the postural analysis criteria of the muscle chains of the Souchard Postural Reeducation method and the plumb line test; and 2) analysis of the type of breathing.

B) Stomatognathic system analysis: 1) evaluation and analysis of the hyoid bone; 2) exploration and evaluation of the termporomandibular joint (TMJ); 3) tongue size and thickness according to the Mallampati classification; 4) analysis of the anatomophysiology of the hard palate, 5) description of the state of the masticatory musculature; 6) classification of occlusion type; and 7) analysis of the movement of the spheno-basilar joint.

A total score is established to determine the greater or lesser risk of suffering obstructive sleep apnea.

## Postural system analysis

In this section, the subject’s postural attitude was analyzed through two observation and measurement tests. Each of them had a different value depending on the findings, and the total sum of the result of all the tests had a higher or lower negative value, in terms of the possibility of suffering from OSA being conditioned by postural alterations. We have included in this area the morphotype analysis, as a novel variable to be evaluated. So that the higher the final score, the higher the risk of suffering from the disease.

### Morphotype analysis

We analyzed the patient’s position in straight bipedalism and carried out the plumb line test with a millimeter reference frame (McGill, 2002; Lafage et al., 2008; Daza Lesmes, 2007). The necessary material to conduct the test was as follows:

- Plumb line.
- Calibration frame.
- Dermic stickers.

The subject was in undergarments and placed in front of the calibration frame, in a side profile position with respect to the observer. The following anatomical references were marked on both sides with body stickers: 1) Lateral edge of the peroneal malleoli; 2) head of the fibula; 3) greater trochanter of the femur; 4) lateral epicondyle; 5) lateral portion of the humeral head; 6) posterosuperior iliac spines (PSIS); 7) anterosuperior iliac spines (ASIS); 8) tragus of the ear.

The subject assumed a reference anatomical position, standing upright, with arms relaxed alongside the body, heels together, 30°-foot opening, and facing forward. The plumb line described a specific path passing in front of the subject’s peroneal malleolus. The position adopted by the plumb line with respect to the other reference points determined the corresponding morphotype, and had a determined value, according to the following description:

Normal Morphotype: Anatomical structures aligned according to the plumb line and reference points = value 0.

Anterior Morphotype: Anatomical references too far forward with respect to the plumb line and reference points = value 1.

Mixed Morphotype: Some anatomical references forward and others backward with respect to the plumb line and reference points = value 2.

Posterior Morphotype: Anatomical references too far backward with respect to the plumb line and reference points = value 3.

Posterior Morphotype with hyperkyphosis of the cervical-thoracic hinge: Anatomical references too far backward with respect to the plumb line and reference points and very marked prominence at the level of C7-T1, C7-T2, or T1-T2 = value of 4. This finding was described by Simon and Travells as anterior head position and is defined by a series of associated anatomical characteristics (Sahrmann et al., 2002; Pilat et al., 2003; Fonseca et al., 2019).

- Hypertonic cervical extensor musculature.
- Alteration at the level of the physiological cervical lordotic curvature, which can give rise to three options: a) increased cervical lordosis up to C4 and kyphosis from C5 to C7; b) global hyperlordotic curve with apex at C5; c) complete cervical lordosis straightening with hyperkyphosis in the cervico-dorsal hinge (C7-T1, T2).
- Hypertension in the occipito-atlantoideal joint, due to occipital hyperextension.
- Hyperelongation of the supra and/or infra hyoid musculature.

### Breathing type analysis

We considered this relevant since the type of oral breathing is related to OSA from very early ages. Several studies determine that mouth-breathing children tend to have apneas, and these persist into adulthood; the altered anatomy and functions in the case of mouth breathers more frequently condition the appearance of OSA (Eckter et al., 2013; Durán et al., 2009). Therefore, we determined whether breathing is nasal or oral, through direct observation of the subject and questions aimed at obtaining this information. In case of doubt, an Altmann mirror was used and place it under the nostrils. The subject was instructed to continue breathing normally, and it was observed whether the mirror fogged up (nasal breather) or not (oral breather). This type of mirror was also used to assess airway and pharyngeal veil alterations (nasal escape) (Durán et al., 2009). We evaluated the findings according to the following criteria:

- Value of 0 = Nasal Breathing.
- Value of 1 = Oral Breathing.

The final result of the score in section A ranged from 0 to 5, with a more negative result indicating a higher final score.

- Value of 0 = No alterations.
- Value of 1 = Low risk of suffering from OSA in relation to postural alterations.
- Value of 2–3 = Moderate risk of suffering from OSA in relation to postural alterations.
- Value of 4–5 = High risk of suffering from OSA in relation to postural alterations.

## Analysis of the stomatognathic system

The following observational and exploratory tests were conducted to specifically evaluate the stomatognathic system. In this area, the position of hyoid bone and the mobility of spheno basilar joint are the novel variables to be evaluated.

### Hyoid position test

Numerous studies have demonstrated that the position of the hyoid bone influences and is influenced by the position of the mandible, head, and cervical spine, and may be affected by different types of occlusion. Its key craniofacial physiological roles are well established, particularly its adaptive response to head and neck postures and movements, given its attachment to the muscles, ligaments, and fascia of the pharynx, mandible, and cranium (Mortazavi et al., 2018).

Hyoid bone position is most frequently assessed radiographically using the S–N plane and the hyoid triangle, defined by the cephalometric landmarks retrognathion (most inferior and posterior point of the mandibular symphysis), hyoidale (most superior and anterior point of the hyoid body), and C3 (most anteroinferior point of the third cervical vertebra). This method relates the hyoid bone to both the cervical spine and the mandible (Espada De-La-Cruz et al., 2021).

Although no consensus exists regarding the relationship between hyoid bone position, tongue posture, and upper airway dimensions across skeletal malocclusions, the literature consistently reports that head and neck movements produce corresponding hyoid displacement. Cephalometric radiographic measurements are considered reliable and reproducible, yet there is no standardized measurement protocol (Mortazavi et al., 2018; Espada De-La-Cruz et al., 2021; Bibby and Preston, 1981).

The evidence supports that the stomatognathic system should be assessed as an integrated functional unit, and that cephalometric analysis methods and the anatomical subdivisions of the pharyngeal airway should be standardized (Mortazavi et al., 2018).

In the present study, our aim is to demonstrate that the hyoid bone moves in concert with the passive head and neck motion applied during the protocol technique. We do not intend to determine its exact position, as radiographic imaging is not included. However, existing evidence suggests that active and/or passive head and neck movement assessment can determine the presence or absence of hyoid mobility, which we propose to correlate with the full set of tests in our protocol (SPOSAP).

The subject was placed in a straight sitting position with the head in a neutral position. The examiner made direct contact with their hyoid bone using interdigital forceps and asks the subject to swallow. Then, they were asked to move their head from side to side and it was determined whether the hyoid bone moves with these movements or not. If the hyoid bone was in a good position, no movement associated with head and neck movements should have been perceived. If, on the other hand, it was in a poor position, movement was perceived when head and neck movements were performed.

Based on the results, the following scoring was given:

- Value of 0 = Well-positioned hyoid bone; no impairment.
- Value of 2 = Descended or ascended hyoid bone, manifest impairment.

### Comprehensive analysis of the temporomandibular joint (TMJ)

First, we conducted a series of guided questions regarding joint pain, headaches, noises and limitation of function (blockage in opening or closing the mouth). After, a series of observational and exploratory tests, based on the criteria of the International Network for orofacial pain and related disorders Methodology, were performed to determine the status of the TMJ.

- Listening: Using a stethoscope and in direct contact with the TMJ, we assessed the presence of clicks, pops, or crepitus. These findings condition the good state of the joint. The result was recorded as positive (if sounds are present) or negative (if no sounds are present).
- Mobility Test: In direct contact with the mandibular condyles, we asked for an active opening and closing of the mouth, and determined if the condyles move symmetrically or asymmetrically, or if there is any other mobility alteration. We recorded the result as positive (if there was asymmetric movement between the mandibular condyles) or negative (if there was symmetrical movement between the mandibular condyles). We also record the alterations in mobility at mouth closure, describing whether there is an S-pattern, C-pattern or normal pattern.
- Pain Test: Whether pain appeared or not upon pressure on the TMJ, taking the mandibular fossa and the joint synovium as references. We also explored the possible points of pain present at the level of the temporal, zygomatic arch and frontal region, according to the recommendations of the International

RDC/TMD Consortium Network (González et al., 2018). We recorded the result as positive (if pain appeared upon pressure) or negative (if no pain appeared upon pressure).

A scale from 0 to two was used according to the following criteria:

- Value of 0 = Normal, joint without impairment. All negative items.
- Value of 1 = Mild impairment, one of the three items to be evaluated is positive.
- Value of 2 = Severe impairment, two or three of the items to be evaluated are positive.

### Tongue size analysis

This was carried out by direct observation of the anatomy of the oral cavity, according to the Friedman scale (or Mallampati modified), considering whether we could see through the mouth opening: 1) the uvula; 2) the faucial isthmus (arches in front of and behind the tonsils); 3) the soft palate. This scale is classically used to determine the difficulty of intubation in patients undergoing surgery and high scores (3 and 4) are also associated with a higher incidence of OSA (Friedman et al., 2017).

A scale from 0 to two was used according to the following criteria:

- Value of 0 = Class I Friedman: total visibility of the tonsils, uvula, and soft palate.
- Value of 1 = Class II Friedman: visibility of the hard and soft palate, upper portion of the tonsils, and uvula.
- Value of 2 = Class III and IV Friedman: hard and soft palate are visible, and the base of the uvula or only the hard palate is visible.

### Analysis of the anatomophysiology of the hard palate

The importance of determining whether there is any alteration at this level is determined by its relationship with the anatomical phenotype associated with OSA. All anatomical factors that reduce the caliber of the UA favor its collapse, and this alteration is key. Abnormal configurations of the hard and soft palate are part of these factors. The high-arched palate is associated with smaller-than-normal cranial bone structures and conditions the collapse of the UA (Dudley et al., 2003). Through direct observation, we assessed findings related to the hard palate.

A scale from 0 to two was used according to the following criteria:

- Value of 0 = Normal; normal palate.
- Value of 1 = Mild impairment; descended palate.
- Value of 2 = Severe impairment; high-arched or twisted palate.

### Analysis of masticatory musculature

This was performed according to an adaptation to the Medical Research Council (MRC) test (González et al., 2018) where values ranged from 0 to 5. In our case we are going to assess muscle tone according to the degree of mouth opening using a scale from 0 to two assessing the degree of active and active-assisted mobility of the temporomandibular joint. They are defined as follows:

- Value 0 = Normal tone, opening and closing of the mouth with no restrictions in mobility.
- Value 1 = Slight hypertonus with no restriction of active TMJ mobility, normal mouth opening.
- Value 2 = Marked hypertonus with restriction of active and active-assisted TMJ mobility, limitation of mouth opening.

The objective was to determine if the masticatory musculature is in optimal condition or, conversely, presented hypo or hypertonicity. Hypertonicity of the masticatory musculature is one of the main features associated with bruxism, which in turn is related to a higher risk of OSA. Around 80% of bruxers manifest apneas at some point in their lives (Via et al., 2013; Ugalde, 2007; Martynowicz et al., 2019; Gutiérrez-Halabi et al., 2022). The buccinator, orbicularis oris, masseter, medial and lateral pterygoids have been analysed.

A scale from 0 to two was used for the assessment of muscle analysis, where:

- Value of 0 = Normal tone.
- Value of 1 = Slight hypertonicity without restriction of active ATM mobility, normal mouth opening and abnormal muscle function.
- Value of 2 = Marked hypertonicity with restriction of active ATM mobility, limitation in mouth opening and abnormal muscle function.

### Classification of the occlusal system

Angle’s classification was followed.

- Class I: Normal occlusion, closure of the upper jaw in front of the lower jaw without protrusion of the upper dental arch.
- Class II 1: Altered occlusion with closure of the upper jaw in front of the lower jaw and marked protrusion of the upper dental arch.
- Class II 2: Altered occlusion with closure of the upper jaw in front of the lower jaw and “edge-to-edge” closure of the upper and lower arches.
- Class III: Altered occlusion with closure of the upper jaw behind the lower jaw and marked retrusion of the upper dental arch. Protrusion of the lower jaw.

Several studies determine that OSA appears more frequently in malocclusion types II2 and III than in cases of type I occlusion (Hernández et al., 2017; Fernández Rodríguez et al., 2008).

A scale from 0 to two was used for the occlusal system class, where:

- Value of 0 = Normal, Class I according to Angle’s classification.
- Value of 1 = Mild impairment, Class II1 according to Angle’s classification.
- Value of 2 = Severe impairment, Class II2 and III according to Angle’s classification.

### Exhaustive evaluation of the spheno-basilar joint

There is no doubt that there is micromobility at the level of the cranial sutures and cranial vault (Sutherland and Magoun 1939); these are rhythmic tensional modifications of conformity at the level of the cranial bones, which are accompanied by rhythmic fluctuations of cerebrospinal fluid, whose main driver is diaphragmatic costal breathing. Thus, changes in rib diameter during a respiratory cycle produce changes in spinal curvatures and in the position of the sacrum, which, through the fascial system and the meninges, pull the occipital bone and bring it to a predetermined position. Hence, sacral flexion produces cranial flexion (via the occipital bone) and spheno-basilar flexion; this movement produces a slight rotation of the sphenoid wings over the sphenoid and the base of the occipital bone (Fernández Rodríguez et al., 2008).

Testing of the Primary Respiratory Movement was performed, in direct contact with the cranial sphere of the subject, to describe the position of the spheno-basilar joint (SBJ), while the subject was asked to take a deep breath. This way, we could conclude whether there was an alteration in the mobility of the SBJ (Busquet et al., 2006; Ricard and Sallé, 2014; Botía et al., 2011).

A scale from 0 to one was used to classify possible alterations of the SBJ, where:

- Value of 0 = Normal, no alteration in SBJ movement.
- Value of 1 = Pathological condition, SBJ in flexion or extension.

The final score for section B ranged from 0 to 13, with a higher score indicating a more negative result.

- Value of 0 = Normal, no alteration
- Value of 1–3 = low impairment, low risk of OSA in relation to the SS.
- Value of 4–7 = moderate impairment, moderate risk of OSA in relation to the SS.
- Value of 8–13 = severe impairment, high risk of OSA in relation to the SS.

The total score for all sections combined ranged from 0 to 18, with a higher score indicating a more negative result. The score was classified according to the following criteria:

- Value of 0 = no apparent risk of OSA.
- Value of 1–3 = low risk of OSA.
- Value of 4–11 = moderate risk of OSA.
- Value of 12–18 = high risk of OSA.

### Statistical analysis

#### Administration and scoring procedure

The SPOSAP protocol is administrated by a trained clinician in a standardized sequence comprising observation, palpation, and functional assessments of both the stomatognathic and postural systems. Each item is scored on a Likert-type scale ranging from 0 (no alteration) to 3 (severe alteration). The total score (TS) is calculated by summing all item scores, with higher values indicating greater dysfunction. Based on the TS, participants can be categorized into three severity levels:

Low dysfunction (TS ≤ 3).

Moderate dysfunction (TS = 4–7).

High dysfunction (TS = 8–13).

(Note: Thresholds should be specified based on validation or expert consensus, if available.)

Standardized administration was ensured by following a detailed protocol manual and training procedures, which contributed to minimizing inter-rater variability.

### Validity

#### Criterion validity

Considering AHI measured by PSG as the gold standard for OSA diagnosis, Spearman correlation test between AHI values, postural system analysis (A), evaluation of the stomatognathic system (B) and the total score of SPOSAP (TS) was conducted for testing the criterion validity.

#### Concurrent validity

Spearman correlation between TS and the variables AHI, ESS, PA, WC, NC, BMI, and ESS was performed. ANCOVA model was conducted by mean difference in variables related with the total score of SPOSAP values controlling for ESS and WC and adjusted by sex and age.

Concurrent validity was examined through Spearman correlation analyses between the total SPOSAP score (TS) and clinically relevant variables. The strongest association was found between TS and AHI (Apnoea–Hypopnoea Index) (r = 0.882, 95% CI [0.812, 0.930], *p* <0.01), supporting the criterion validity of the protocol. TS also showed significant positive correlations with:

- Epworth Sleepiness Scale (ESS): r = 0.552, 95% CI [0.373, 0.683], *p* <0.01
- Waist circumference (WC): r = 0.305, 95% CI [0.105, 0.478], *p* <0.01
- BMI: r = 0.204, 95% CI [0.011, 0.389], *p* <0.05

This model allowed us to assess whether the mean values of the dependent variables varied significantly across OSA risk groups, while adjusting for age and sex. The inclusion of these covariates was based on their known association with both OSA risk and the clinical outcomes measured. Post hoc comparisons were performed using Bonferroni correction to control for multiple testing.

#### Reliability

Test-retest reliability was examined in a subsample of 10 subjects 3 weeks after the initial measurement. The tests-retest intraclass correlation coefficient was used in the reproducibility analysis of the SPOSAP.

The results of all applied tests were considered significant with a p-value <0.01; p value < 0.05 and a confidence interval (CI) of 95%. SPSS statistical software version 29.0.1.0 (171) was used for data analysis and processing.

The internal consistency of the SPOSAP protocol was assessed using Cronbach’s alpha, yielding a value of 0.89, which indicates excellent reliability. Item-total correlations ranged from 0.61 to 0.78, demonstrating good coherence among components of the protocol. The removal of individual items did not significantly improve the alpha coefficient, supporting the internal stability of the full scale.

These results indicate that the SPOSAP total score aligns with variables commonly associated with OSA, supporting its concurrent and construct validity.

All correlations were tested using a two-tailed significance level of 0.05, and 95% confidence intervals were computed using bootstrapping (1000 samples) to enhance robustness.

A *post hoc* power analysis was performed using G*Power 3.1. For the primary correlation between the SPOSAP total score and the Apnoea–Hypopnoea Index (r = 0.882), with a significance level of 0.05 and a total sample size of 105 participants, the achieved statistical power was greater than 0.99.

## Results

A total of 105 patients were evaluated during the data collection and measurement period, with participant ages ranging from 23 to 83 years (m = 52.25; SD = 12.22), and 65% of the sample being male (66 participants). A total of 68 participants (64.7% of the total sample) were diagnosed with varying levels of OSA. Of the whole sample, 20.6% (14 participants) had mild OSA, 27.9% (19 participants) had moderate OSA, 51.5% (35 participants) had severe OSA, and 35.3% (37 participants) were not diagnosed at the time of evaluation but reported at least two symptoms ± related to the condition. All characteristics of the study population are detailed in Table 1.

**TABLE 1:** Sociodemographic characteristics of population.

	Diagnosed population n = 68 mean ± SD	Non diagnosed population n = 37 mean ± SD	P Value
Age	53.71 ± 11.91	51.05 ± 11.44	0.505
Gender (%)	men = 64 women = 35.3	men = 56.8 women = 43.2	0.423
Occupation (%)	assets = 81.1 inactive = 19.1	assets = 80.9 inactive = 19.1	0.980
Blood pressure (%)	normotensive = 41.2 hypertensive = 58.8	normotensive = 59.5 hypertensive = 40.5	0.073
Physical activity (%)	WHO = 15 active = 23 inactive = 30	WHO = 37.8 active = 35.1 inactive = 27	0.135
BMI	28.68 ± 3.86	27.35 ± 2.96	0.347
WC	110.58 ± 18.45	100.91 ± 15.55	0.486
NC	41.48 ± 5.19	40.79 ± 4.27	0.250
ESS	9.34 ± 4.66	8.14 ± 3.30	0.040**
AHI value	56.70 ± 33.77		<0.01**
Mild OSA (%)	20.6		
Moderate OSA (%)	27.9		
Severe OSA (%)	51.5		
No diagnosed (%)		35.3	

BMI, body mass index; WC, waist circumference; NC, neck circumference; ESS, epworth sleepiness scale; AHI, apnea and hypopnea index; OSA, obstructive sleep apnea; n, population; WHO, active people according to the recommendations of the World Health Organization; active, active population but not according to World Health Organization recommendations; inactive, non active population.

All statistical analyses were conducted using IBM SPSS Statistics, version 29.0.2.0 (IBM Corp., Armonk, NY). Descriptive statistics are presented as means ± standard deviations (SD) for continuous variables and as absolute and relative frequencies for categorical variables. Group comparisons (diagnosed vs. non-diagnosed; low, medium, high risk of OSA) were performed using independent-samples tests and one-way ANCOVA models as appropriate. For ANCOVA, age and sex were included as covariates to adjust for their potential confounding effects. Post hoc comparisons were corrected using the Bonferroni method to control the family wise error rate. Spearman correlation analyses were performed to assess the relationships between the Apnoea–Hypopnoea Index (AHI), total protocol score (TS), postural system evaluation (A), stomatognathic system evaluation (B), and other variables such as BMI, waist circumference (WC), neck circumference (NC), Epworth Sleepiness Scale (ESS), and physical activity (PA). Only correlations significant at the 0.05 or 0.01 level were considered. Potential confounding factors—such as age, sex, BMI, blood pressure, and PA—were identified *a priori* and accounted for either through statistical adjustment or stratification. No imputation was necessary, as missing data were minimal; analyses were conducted using complete-case data. Sensitivity analyses were performed by replicating key findings across OSA risk categories and adjusting models to test the robustness of associations. All tests were two-tailed and a p-value <0.05 was considered statistically significant.

### Criterion validity

To assess criterion validity of the protocol we analyzed a total of 68 participants had AHI by PSG. The analysis of the correlation between the total score (TS) and the AHI, as well as with relevant clinical and anthropometric variables was highly relevant. A strong and statistically significant positive correlation was observed between TS and AHI (r = 0.882; p < 0.01), supporting the protocol´s criterion validity (Table 2).

**TABLE 2:** Relationship between final protocol score, AHI value, postural system analysis and evaluation of stomatognathic system.

	TS	A	B
AHI	0.882**	0.707**	0.880**
TS		0.824**	0.907**
A			0.646**

AHI, apnea and hypopnea index; A, postural system analysis; B, evaluation of the stomatognathic system; TS, total score of protocol.

Note: *. Correlation is significant ** at the 0.01 level (bilateral).

### Concurrent validity

In addition, TS showed significant concurrent correlations with ESS (r = 0.552; p < 0.01), waist circumference (WC) (r = 0.305; p < 0.01), and BMI (r = 0.204; p < 0.05), indicating that higher protocol scores are meaningfully associated with known OSA-related parameters (Table 3).

**TABLE 3:** Relationship between apnea and hypopnea index, total score of the protocol, waist circumference, neck circumference, physical activity, body mass index and Epworth Sleepiness Scale.

	TS	PA	BMI	WC	NC	ESS
AHI	0.882**	0.043	0.188	0.372**	0.282*	0.627**
TS		0.134	0.204	0.305**	0.131	0.552**
PA			0.159	0.124	0.055	0.193*
BMI				0.830**	0.480**	0.201*
WC					0.473**	0.244*
NC						0.009

Note: *. Correlation is significant *at the 0.05 level (bilateral) and ** at the 0.01 level (bilateral).

AHI, apnea and hypopnea index; BMI, body mass index; ESS, epworth sleepiness scale; NC, neck circumference; PA, physical activity; TS, total score; WC, waist circumference.

Based on the results of the concurrent analysis, an ANCOVA analysis was performed between the statistically significant variables (ESS and WC) and the different levels of risk of suffering the disease according to SPOSAP, controlling for potential confounding factors according to age and waist circumference. The results of the analysis indicate that the ESS variable is statistically significant for discriminating between the three SPOSAP risk levels (mild, medium and high) and that the WC variable is statistically significant for discriminating between the mild and high levels of risk of disease according to SPOSAP (Table 4).

**TABLE 4:** Mean difference in variables related with OSA by Protocol values controlling by age and sex (ANCOVA model).

	Low risk of OSA n = 26	Medium risk of OSA n = 39	High risk of OSA n = 40	P	Post hoc
ESS	4.77 ± 3.51	9.08 ± 2.65	11.45 ± 3.92	<0.01	L < M < H
WC	104.93 ± 19.43	100.71 ± 15.41	114.93 ± 16.88	<0.03	L < M < H

Values are means standard deviation (SD). L, low (AHI<15); M, medium (AHI 〈30〉15); H, high (AHI>30); Post Hoc hypothesis tests determined with the Bonferroni correlation for multiple comparison, p value <0.01 or <0.05; ns, non-significant.

ESS, epworth sleepiness scale; n, population; WC, waist circumference; p, significant.

### Reliabilty

To assess the internal consistency of the scale, Cronbach’s alpha coefficient was calculated, and the result has been positive (0.926). This result confirms the reliability of the questionnaire if it is above 0.70 in cases where the same measurements are repeated on the same subjects within a short period of time. The test–retest reliability was assessed in a subsample (n = 10) using the intraclass correlation coefficient (ICC), yielding a value of >90%. However, due to the small sample size, these results should be interpreted with caution and considered exploratory. All the tests of the assessment protocol were repeated, except the PSG in the cases already diagnosed, the first measurement was carried out at the beginning of the study, when they were invited to participate in the study, and the second one 3 weeks later. The test-retest intraclass ratio coefficient was used in the reproducibility analysis of the SPOSAP. The results of all applied tests were considered significant with a p-value <0.01, p-value <0.05 and a confidence interval (CI) of 95%.

## Discussion

The main objective of this study was to confirm the validity and reliability of a new measurement instrument for people not diagnosed with OSA but with compatible symptoms. The statistical approach employed in this study was specifically designed to align with the main objective. The analyses provided robust psychometric evidence supporting the protocol’s clinical relevance.

First, the high internal consistency (Cronbach’s alpha = 0.89) supports the reliability of the SPOSAP protocol as a cohesive instrument. The positive and statistically significant correlations between the total score (TS) and clinical markers such as AHI (r = 0.882), ESS (r = 0.552), BMI, and waist circumference further confirm its concurrent validity and its ability to reflect established OSA-related variables.

Importantly, the ANCOVA models demonstrated the protocol’s capacity to distinguish between different levels of OSA severity, even after adjusting for age and sex. These findings are consistent with the theoretical rationale of SPOSAP, which integrates postural and stomatognathic assessments—two domains often considered separately in OSA screening tools. The ability of the protocol to capture morphophysiological and postural alterations associated with OSA supports the hypothesis that posture plays a meaningful role in both the identification and potential management of the disorder. This highlights the clinical potential of incorporating postural evaluation into routine screening and of expanding Myofunctional Therapy (MT) to include interventions targeting muscle chains and postural control. As such, SPOSAP offers a more integrative approach to both diagnosis and intervention planning in this population.

After analysis of the results and statistical tests, we can say that the SPOSAP is a valid and reliable instrument for assessing the risk of OSA. In the systematic review conducted prior to this study, aimed at examining the available scientific literature where posture and its possible alterations were considered within interventions for OSA, only one study was found where postural therapy was included in the intervention, and it was used as a complement to MT. The results obtained in said study were promising (Hernández et al., 2017). MT has been used for years in the treatment of OSA, focusing on improving the condition of structures belonging to the SS and involving these structures. Until now, postural therapy had not been included in the approach with MT, despite being considered an integral part of the orofacial and cranial complex and determining its possible alterations. Also, as regard sleep, there are studies proposing therapeutic alternatives applied during sleep to modify posture, devices that limit the supine position for as long as possible with the aim of improving apnea and hypopnea indicators (Atilgan et al., 2019; Camacho et al., 2015; Mackay et al., 2020; Ng and Yow, 2019). This fact is related to the differences produced in the UA, especially in the pharynx, when one or the other position is adopted (supine decubitus-lateral decubitus). Studies have conducted during both sleep and wakefulness, confirming anatomical alterations in the subjects in both periods, which shows that posture conditions functional alterations that are determinant in OSA and in the manifestation of its symptoms (Ng and Yow, 2019; Ravesloot et al., 2017). However, in recent years, there is an increasing trend towards reducing the number of hours of sleep and worsening its quality, which increases the risk of developing sleep disorders, with a higher prevalence in the general population (Yalamanchili et al., 2019; Kastoer et al., 2018). Nonetheless, a large part of the population remains undiagnosed, which is due to factors such as difficult access to diagnostic methods and the limited perception of sleep symptoms as a problem, or at least as a problem of severity (Beddis et al., 2018; Zancanella et al., 2014; Bittencourt and Caixeta, 2010). In the case of our protocol, we analysed a sample of 105 subjects, the sample is divided into subjects diagnosed (n = 68) and undiagnosed but with symptoms compatible with OSA (n = 37). The sample is consistent with the type of population in which OSA is most prevalent, men over 50 years of age (mean age = 52.25); percentage of men in the sample: 65%. The variables included in the assessment protocol have already been used in numerous previous studies and are considered in different interventions: a) termporomandibular joint (TMJ); b) tongue; c) oral breathing habit; d) palate; e) masticatory muscles; f) occlusal type. In addition, three new items are included that, until now, had not been related or evaluated within the SS analyses for OSA: a) Morphotype; b) Spheno-basilar joint (SBJ); c) Hyoid bone (HB). The inclusion of these new variables in the specific assessment of OSA adds value to the protocol and provides a broader view of all the aspects that may influence the manifestations of the pathology and its related symptoms. It offers a more comprehensive view of the subject, and includes morphological characteristics that we believe cannot be overlooked in the complete analysis of SS. In addition, the final score of the protocol correlates very well with the AHI, and using the ESS as a complement, it allows us to classify people without a diagnosis but with compatible symptomatology, with a mild, moderate, or severe risk of suffering the disease. The early use of this assessment protocol in patients with symptoms compatible with OSA or with specific anatomical and morphological features can be a very useful tool for screening certain populations to improve early diagnosis. The SPOSAP offers a quantitative analysis of the evaluation tests, this information can be easily interpreted, both in the initial evaluation and in subsequent re-evaluations and improves the understanding of the findings in a multidisciplinary intervention team. This allows us to confirm that there may be a direct relationship between postural alterations and obstructive sleep apnea, and that the anatomical elements (hyoid and basilar sphenoid) should be considered, as they reinforce the hypothesis of the relationship with the rest of the anatomical structure. With the use of this assessment tool, we can easily analyse all the variables, define them, and integrate them in the overall assessment of the subjects and in the subsequent treatment. The SPOSAP thus joins the most recent therapeutic trends in OSA, which propose different multidisciplinary options and seek to obtain the best results in the management of this underdiagnosed and increasingly prevalent disease.

Although the recruitment strategy involved multiple channels, including public announcements, hospital-based infographics, and database searches, which may introduce a certain degree of selection bias, this approach was chosen to ensure an adequate sample size and capture a broad spectrum of individuals with symptoms compatible with OSA. As a result, the sample presented a wide age range (23–83 years) and clinical heterogeneity, reflecting the real-world variability observed in clinical practice. To address the potential influence of these factors on the results, additional stratified and multivariate analyses were conducted by age, sex, and BMI. These analyses confirmed the robustness of the main findings, although the smaller subgroup sizes inevitably reduced the statistical power. This limitation has been acknowledged, and future studies with larger, more homogeneous samples are warranted to further validate these findings.

Therefore, these results should be interpreted with caution. We believe that more studies of this type should be carried out, and in other types of populations, mainly in the pediatric population, as many of the findings may be less relevant during childhood and have a better response to interventions. Additionally, in said population, comorbidities are less frequent, which enhances the success of the results in the different intervention therapies.

## Potencial limitations

This study has several methodological limitations that should be considered when interpreting the findings. First, the relatively small sample size and single-center design may limit the generalizability of the results to broader populations. Second, the cross-sectional nature of the study prevents conclusions about causality or long-term effects. Third, although we employed a validated and standardized protocol to minimize measurement bias, the absence of a control group may reduce the strength of comparisons. The associations observed in the present study do not allow for the establishment of causal relationships between postural alterations and OSA. It therefore remains uncertain whether these alterations act as causal factors, contribute to the aggravation of the disorder, or arise as a consequence of it. This limitation is partly attributable to the absence of a control group composed of healthy subjects without OSA symptoms, which would have enabled more definitive comparative analyses Additionally, despite efforts to control for potential confounders, unmeasured variables such as physical activity levels, psychological stress, or unreported comorbidities could have influenced the outcomes. Future studies with larger, more diverse samples and longitudinal designs are warranted to confirm and extend these findings.

## Conclusion

According to the results obtained in the study and the analysis of the statistical data, we can say that the SPOSAP can be a useful screening tool for the early diagnosis of patients with symptoms compatible with OSA and expand the current approach of new multidisciplinary treatment trends for this pathology. We believe it provides a comprehensive approach to the multifactorial manifestations present in patients with OSA, and we can be more specific in its management, improving the present symptomatology and probably the adherence to more classical treatments.

## Data Availability

The raw data supporting the conclusions of this article will be made available by the authors, without undue reservation.
