# Correction: Validity and reliability of an protocol of the stomatognathic and postural system evaluation for patients with obstructive sleep apnea: a cross-sectional study

**DOI:** 10.3389/fphys.2025.1755604

**Published:** 2025-12-19

**Authors:** Marta Guijarro-Herraiz, Natalia M. Arias Palencia, Maria Figueroa Mayordomo, Rocío Palomo Carrión, Blanca Notario Pacheco

**Affiliations:** 1 Faculty of Nursing, Physiotherapy and Occupational Therapy, University of Castilla-La Mancha, Toledo, Spain; 2 Department of Physiotherapy, Faculty of Health Sciences, Universidad Europea de Valencia, Valencia, Spain; 3 Department of Nursing, Faculty of Physiotherapy and Nursing, Physiotherapy and Occupational Therapy, ImproveLab Research Group, University of Castilla-La Mancha, Toledo, Spain; 4 Department of Nursing, Faculty of Nursing of Cuenca, Physiotherapy and Occupational Therapy, University of Castilla-La Mancha, Cuenca, Spain; 5 Faculty of Education of Cuenca, University of Castilla-La Mancha, Cuenca, Spain

**Keywords:** obstructive sleep apnea, myofunctional therapy, sleep apnea, postural reeducation, stomatognathic system, oropharyngeal exercises

Author Marta Guijarro-Herraiz was erroneously assigned to affiliations Hospital Virgen de la Luz, Cuenca, Spain and Facultad de Enfermería, Fisioterapia y Podología, Universidad Complutense de Madrid, Madrid, Spain. These affiliation have now been removed for author Marta Guijarro Herraiz.

The correct affiliation for author Marta Guijarro-Herraiz is Faculty of Nursing, Physiotherapy and Occupational Therapy, University of Castilla-La Mancha, Toledo, Spain.

The original article has been updated.

