# Supplementary material for: Validity and reliability of an protocol of the stomatognathic and postural system evaluation for patients with obstructive sleep apnea: a cross-sectional study
Source: Front Physiol. 2025 Sep 30;16:1649593. doi: 10.3389/fphys.2025.1649593 (PMC12518225; doi:10.3389/fphys.2025.1649593)
Supplement: Supplementary file 1 [file DataSheet1.pdf]

Table 1.- Sociodemographic characteristics of population.

	Diagnosed population n=68 *Mean ± SD*	*Non diagnosed population* n=37 *Mean ± SD*	*p value*
Age	53.71± 11.91	51.05±11.44	0.505
Gender (%)	men=64<br>women=35.3	men=56.8<br>women=43.2	0.423
Occupation (%)	assets=81.1<br>inactive=19.1	assets=80.9<br>inactive=19.1	0.980
Blood pressure (%)	normotensive = 41.2<br>hypertensive= 58.8	normotensive = 59.5<br>hypertensive= 40.5	0.073
Physical activity (%)	WHO= 15<br>active = 23<br>inactive = 30	WHO= 37.8<br>active =35.1<br>inactive = 27	0.135
BMI	28.68±3.86	27.35±2.96	0.347
WC	110.58±18.45	100.91±15.55	0.486
NC	41.48±5.19	40.79±4.27	0.250
ESS	9.34±4.66	8.14±3.30	0.040**
AHI value	56.70±33.77		< 0.01**
Mild OSA (%)	20.6		
Moderate OSA (%)	27.9		
Severe OSA (%)	51.5		
No diagnosed (%)		35.3	

BMI: Body mass index; WC = waist circumference; NC = neck circumference; ESS = Epworth Sleepiness Scale; AHI=apnea and hypopnea index; OSA = obstructive sleep apnea; n=population; WHO = active people according to the recommendations of the World Health Organization; active= active population but not according to World Health Organization recommendations; inactive = non active population.

Table 2.- Relationship between final protocol score, AHI value, postural system analysis and evaluation of stomatognathic system.

	TS	A	B
**AHI**	0.882**	0.707**	0.880**
**TS**		0.824**	0.907**
**A**			0.646**

AHI= apnea and hypopnea index; A=postural system analysis; B= evaluation of the stomatognathic system; TS = total score of protocol.

*Note: *. Correlation is significant** at the 0.01 level (bilateral)*

Table 3.- Relationship between apnea and hypopnea index, total score of the protocol, waist circumference, neck circumference, physical activity, body mass index and Epworth Sleepiness Scale.

	TS	PA	BMI	WC	NC	ESS
**AHI**	0,882**	0.043	0.188	0.372**	0.282*	0.627**
**TS**		0.134	0.204	0.305**	0.131	0,552**
**PA**			0.159	0.124	0.055	0.193*
**BMI**				0.830**	0.480**	0.201*
**WC**					0.473**	0.244*
**NC**						0.009

*Note: *. Correlation is significant *at the 0.05 level (bilateral) and ** at the 0.01 level (bilateral)*

AHI=apnea and hypopnea index; BMI=body mass index; ESS=Epworth sleepiness scale; NC=neck circumference; PA=physical activity; TS=total score; WC = waist circumference.

Table 4.- Mean difference in variables related with OSA by Protocol values controlling by age and sex (ANCOVA model)

	LOW RISK OF OSA n=26	MEDIUM RISK OF OSA n=39	HIGH RISK OF OSA n=40	p	Post Hoc
ESS	4.77±3.51	9.08±2.65	11.45±3.92	<0.01	L<M<H
WC	104.93±19.43	100.71±15.41	114.93±16.88	<0.03	L<M<H

Values are means±standard deviation (SD). L, low (AHI<15); M, medium (AHI <30>15); H, high (AHI>30); Post Hoc hypothesis tests determined with the Bonferroni correlation for multiple comparison, p value < 0.01 or <0.05; ns= non-significant.

ESS = Epworth Sleepiness Scale; n = population; WC= waist circumference; p=signification.

Tabla 5.- Correlations (criterion and concurrent validity) with your overall data

**Variable**	**Diagnosed mean±SD**	**Non-Diagnosed mean±SD**	**p**
**Age (years)**	53.71±11.91	51.05±11.44	0,505
**Gender(%M/W)**	64/35.3	56.8/43.2	0,423
**Occupation (%ASS/I))**	81.1/19.1	80.9//19.1	0,98
**Blood pressure (%N/HT)**	41.2/58.8	59.5/40.5	0,073
**Physical activity(%WHO/A/I)**	15/23/30	37.8/35.1/27	0,135
**BMI**	28.68±3.86	27.35±2.96	0,347
**WC**	110.58±18.45	100.91±15.55	0,486
**NC**	41.48±5.19	40.79±4.27	0,25
**ESS**	9.34±4.66	8.14±3.30	0,04
**AHI value**	56.70±33.77	-	<0,01

A = active; AHI = apnea and hipoapnea index; ASS = assets; BMI = body mass index; ESS = Epworth Sleepiness Scale; HT = hypertensive; I = inactive; M = men; N = normotensive; NC = neck circumference; WC = waist circumference; W = women; WHO = criteria of World Health Organization

Tabla 6.- Supplementary material on sample descriptions

Variable	n_total	rho_total	p_total
AHI vs TS	105	0,882	<0.01
AHI vs A	105	0,707	<0.01
AHI vs B	105	0,88	<0.01
TS vs A	105	0,824	<0.01
TS vs B	105	0,907	<0.01
A vs B	105	0,646	<0.01
AHI vs PA	105	0,043	ns
AHI vs BMI	105	0,188	ns
AHI vs WC	105	0,372	<0.01
AHI vs NC	105	0,282	<0.05
AHI vs ESS	105	0,627	<0.01
TS vs PA	105	0,134	ns
TS vs BMI	105	0,204	ns
TS vs WC	105	0,305	<0.01
TS vs NC	105	0,131	ns
TS vs ESS	105	0,552	<0.01
PA vs BMI	105	0,159	ns
PA vs WC	105	0,124	ns
PA vs NC	105	0,055	ns
PA vs ESS	105	0,193	<0.05
BMI vs WC	105	0,83	<0.01
BMI vs NC	105	0,48	<0.01
BMI vs ESS	105	0,201	<0.05
WC vs NC	105	0,473	<0.01
WC vs ESS	105	0,244	<0.05
NC vs ESS	105	0,009	ns

A= group A tests; AHI = apnea and hipoapnea index; B= group B tests; BMI=body. Mass index; ESS = Epworth Sleepiness Scale; NC= neck circumference; PA=Physical activity; TS = total score; WC = waist circumference.
